# Sertoli Cell-Specific Deletion of *Hsp90aa1* Does Not Overtly Impair Male Fertility in Mice

**DOI:** 10.3390/cells15211962

**Published:** 2026-10-28

**Authors:** Sunjiangnan Wu, Linggeng Kong, Miao Zhu, Bing Yao, Shanmeizi Zhao, Li Chen

**Affiliations:** 1 State Key Laboratory of Reproductive Medicine and Offspring Health, Nanjing Medical University, Nanjing 211166, China; jiangnan_ws@stu.njmu.edu.cn (S.W.); yaobing@nju.edu.cn (B.Y.); 2 Department of Reproductive Medicine, Affiliated Jinling Hospital, Medical School, Nanjing University, Nanjing 210002, China; 3 Department of Chemistry, University of Hong Kong, Hong Kong, China; u3679556@connect.hku.hk; 4 Department of Reproductive Medicine, Jinling Clinical Medical College, Nanjing University of Chinese Medicine, Nanjing 210023, China; zhumiao9707@163.com

**Keywords:** HSP90α, *Hsp90aa1*, Sertoli cells, spermatocytes, male fertility, conditional knockout

## Abstract

**Highlights:**

**What are the main findings?**

- Sertoli-cell HSP90α was markedly reduced, but CKO males remained fertile.
- Spermatocyte counts declined modestly, while sperm parameters were preserved.

**What are the implications of the main findings?**

- Sertoli-cell HSP90α is not essential for maintaining fertility under basal conditions.
- HSP90α requirements in male reproduction are cell type-specific.

**Abstract:**

The stress-inducible molecular chaperone HSP90α, encoded by *Hsp90aa1*, plays an essential role in male reproduction. Systemic *Hsp90aa1* deficiency causes pachytene arrest, germ cell loss, and azoospermia. However, systemic models cannot distinguish effects originating in germ cells from those mediated by the testicular somatic microenvironment. Sertoli cells sustain germ cell development through physical support, nutrient supply and regulatory signaling. The function of HSP90α in Sertoli cells has previously been studied mainly in cultured cells, with a lack of in vivo evidence. To investigate its role in Sertoli cells, we generated *Hsp90aa1^flox/flox^*; *Amhr2-Cre*^+^ conditional knockout (CKO) mice. HSP90α was markedly reduced in Sertoli cell-enriched fractions from CKO testes, and testicular immunofluorescence showed reduced signal in Sertoli cell regions. Despite this reduction, CKO males remained fertile and showed no significant abnormalities in testicular morphology, spermatogenic progression, the measured sperm parameters, Sertoli cell counts, or the localization of the junction-associated proteins. However, total germ cell counts were modestly reduced in CKO testes, with the reduction primarily observed in spermatocytes. These findings indicate that Sertoli-cell *Hsp90aa1* deletion is associated with a modest reduction in spermatocyte numbers but does not overtly impair male fertility under the conditions examined. The contrast with systemic *Hsp90aa1* deficiency suggests that the requirement for HSP90α in male reproduction is cell type-specific.

## 1. Introduction

Heat shock protein 90 (HSP90) is a highly conserved ATP-dependent molecular chaperone that assists the folding and maturation of diverse client proteins and helps maintain proteostasis [1]. It also regulates signal transduction, metabolism, and cellular stress responses [1]. Mammalian cells contain two major cytosolic HSP90 isoforms with distinct expression patterns. HSP90α is encoded by *Hsp90aa1* and induced by stress, whereas HSP90β is encoded by *Hsp90ab1* and expressed constitutively [2]. Genetic studies have established an essential role for HSP90α in male reproduction. Global disruption of *Hsp90aa1* prevents spermatocytes from progressing beyond the pachytene stage, resulting in the absence of mature spermatozoa and male infertility [3]. Similarly, tamoxifen-inducible deletion of *Hsp90aa1* in adult mice causes extensive germ cell loss and spermatogenic arrest [4]. HSP90α also contributes to piRNA biogenesis and retrotransposon repression in male germ cells [5]. Thus, HSP90α is required for regulating spermatogenesis. However, because these systemic models disrupt *Hsp90aa1* in multiple tissues and testicular cell populations, they cannot determine whether the resulting infertility arises solely from defects in germ cells or also from disruption of the testicular somatic microenvironment.

Among the testicular somatic cell types that could contribute to the infertility caused by systemic *Hsp90aa1* deficiency, Sertoli cells are particularly relevant because they establish and maintain the microenvironment in which germ cells develop [6,7]. Within the seminiferous epithelium, they directly contact developing germ cells, supplying nutrients and structural support [6,7] and transmitting paracrine signals [8,9]. They also establish the blood–testis barrier (BTB), a dynamic junctional complex comprising cytoplasmic adaptor protein ZO-1 and transmembrane tight junction protein occludin [10,11]. The localization of junction-associated proteins changes across the seminiferous epithelial cycle as the BTB undergoes remodeling [10,12]. Disruption of Sertoli cell junctions can impair spermatogenesis [13,14,15,16,17]. Sertoli cells also respond to endocrine and local signals that regulate their development and support of germ cells [8,18,19]. These activities depend on the regulation of numerous proteins. Given the established role of HSP90 in client-protein maturation and stabilization [1], HSP90α may therefore contribute to Sertoli cell function and the spermatogenic microenvironment.

Direct evidence linking HSP90α to Sertoli cell function remains limited. In cultured immature porcine Sertoli cells, *HSP90AA1* overexpression increased cell viability, lactate dehydrogenase A activity, and lactate production, whereas treatment with the HSP90 inhibitor 17-AAG reduced these measures and promoted apoptosis [20]. These findings suggest that HSP90 activity affects Sertoli cell survival and metabolism in vitro. However, evidence from an immature porcine cell culture model and pharmacological inhibition cannot establish whether endogenous HSP90α is physiologically required in Sertoli cells within the intact testis. A cell-specific genetic model is therefore needed to define its function in vivo.

To address this question, we established an in vivo model via *Amhr2*-Cre to delete *Hsp90aa1* in Sertoli cells and examined postnatal testicular development and male fertility. CKO males remained fertile and showed no major abnormalities in the reproductive measures examined, although germ cell counts were modestly reduced, primarily among spermatocytes. The absence of the severe phenotype observed after systemic *Hsp90aa1* loss suggests that HSP90α has distinct contributions across testicular cell populations.

## 2. Materials and Methods

### 2.1. Animals

The constitutive *Amhr2*-Cre line was provided by Professor Jianmin Li at the Animal Core Facility of Nanjing Medical University. The *Hsp90aa1*-floxed mice (Shanghai Model Organisms Center, Shanghai, China, NM-CKO-220168) were crossed with the *Amhr2*-Cre line. Male *Hsp90aa1^flox/flox^*; *Amhr2-Cre*^+^ mice were designated as the conditional knockout (CKO) group. Male *Hsp90aa1 ^flox/flox^*; *Amhr2-Cre*^−^ littermates served as controls. All mice had a C57BL/6 genetic background. They were housed in a specific pathogen-free facility under a 12 h light/12 h dark cycle. The room temperature was maintained at 20–22 °C, and the relative humidity was 50–70%. The Institutional Animal Care and Use Committee of Nanjing Medical University approved all animal procedures (Approval No. IACUC-2401029). The study followed the institutional guidelines for laboratory animal care and use. Fertility testing began when the males reached 8 weeks of age. Each control or CKO male was housed with two fertile adult wild-type (WT) females for 2 months. For each male, we recorded the total number of litters and the number of pups in each litter.

### 2.2. Genotyping

For routine genotyping, toe samples were collected from mice at postnatal day 7. Genomic DNA was prepared using the One Step Mouse Genotyping Kit (Vazyme, Nanjing, China, PD101-01). PCR was performed with primers for the *Hsp90aa1-flox* and *Amhr2-Cre* alleles. The amplified products were separated on agarose gels and photographed with a gel imaging system. Primer sequences for genotyping and RT-qPCR are listed in Table 1.

### 2.3. Analysis of Publicly Available Single-Cell RNA Sequencing Data

We used the GSE112393 single-cell RNA sequencing dataset generated by Green et al. [21] to examine *Hsp90aa1* transcripts across annotated cell populations in adult mouse testes. We viewed the processed data in SMARTdb [22]. The violin plot was generated directly in SMARTdb. We did not further process the data or perform statistical comparisons between cell populations.

### 2.4. Cell Culture, Primary Testicular Cell Isolation, and Assessment of Sertoli Cell Enrichment

GC-2spd(ts) cells (Procell Life Science & Technology, Wuhan, China, CL-0593) and TM4 cells (Procell Life Science & Technology, Wuhan, China, CL-0456) were cultured in DMEM/F12 (BasalMedia, Shanghai, China, L310KJ) containing 10% fetal bovine serum (HAKATA, Shanghai, China, HN-FBS-50) and 1% penicillin–streptomycin (Beyotime, Shanghai, China, C0224). Both cell lines were maintained at 37 °C in a humidified incubator with 5% CO_2_.

Primary testicular cells were isolated from 3-week-old male mice using a procedure described in our previous publications [23,24]. For each isolation, testes from 3 mice of the same genotype were pooled. After removal of the tunica albuginea, seminiferous tubules were sequentially digested at 37 °C with 0.25% trypsin (Gibco, Grand Island, NY, USA, 15050-065) for 5 min, followed by 1 mg/mL collagenase I (Worthington, Lakewood, NJ, USA, LS004194) for 7 min. The cell suspension was filtered through a 100 μm cell strainer (Corning, Corning, NY, USA, 352360) and plated in the complete DMEM/F12 medium described above. Primary cells were maintained at 34 °C in a humidified incubator with 5% CO_2_. After 4 h, nonadherent cells were collected as the germ cell-enriched fraction, whereas the adherent Sertoli cell-enriched fraction was cultured for 4 days under the same conditions before collection. The composition of the adherent fraction was evaluated by immunofluorescence staining for the Sertoli cell markers SOX9 and vimentin [25,26], using the primary antibodies listed in Table 2. Nuclei were counterstained with DAPI-containing mounting medium (Sigma-Aldrich, St. Louis, MO, USA, F6057). For each isolation and each marker, 1000 DAPI-stained nuclei were evaluated. The proportion of marker-positive cells was calculated by dividing the number of marker-positive cells by the total number of DAPI-stained nuclei.

### 2.5. RNA Extraction and Quantitative Real-Time PCR

Total RNA was extracted from cell samples using the AFTSpin Tissue/Cell Fast RNA Extraction Kit (ABclonal, Wuhan, China, RK30120). Equal amounts of RNA from each sample were reverse-transcribed into cDNA with ABScript Neo RT Master Mix for qPCR with gDNA Remover (ABclonal, Wuhan, China, RK20433). Quantitative real-time PCR was carried out on a LightCycler 96 Real-Time PCR System (Roche, Mannheim, Germany) using BrightCycle Blue Universal SYBR Green qPCR Mix with UDG (ABclonal, Wuhan, China, RK21220). Relative *Hsp90aa1* expression was normalized to *Gapdh* and calculated using the 2^−ΔΔCt^ method [27]. Three technical replicates were performed for each biological sample. Primer sequences are listed in Table 1.

### 2.6. Protein Extraction and Western Blotting

Cell pellets or testicular tissues were lysed on ice in RIPA lysis buffer (Thermo Fisher Scientific, Waltham, MA, USA, 89901) supplemented with a protease inhibitor cocktail (Med Chem Express, Monmouth Junction, NJ, USA, HY-K0011) and PMSF (Beyotime, Shanghai, China, ST506). Protein concentration was measured using the Pierce BCA Protein Assay Kit (Thermo Fisher Scientific, Waltham, MA, USA, 23225). Equal amounts of protein from each sample were separated on 8% SDS-PAGE and subsequently transferred onto a PVDF membrane. Membranes were blocked with 5% BSA in TBST for 1 h at room temperature and incubated overnight at 4 °C with the appropriate primary antibodies listed in Table 2. After washing, the membranes were incubated with the corresponding HRP-conjugated secondary antibodies for 1 h at room temperature. Protein bands were visualized using an ECL chemiluminescence substrate (Vazyme, Nanjing, China, E422) and imaged using a Tanon-5200 imaging system (Tanon, Shanghai, China).

### 2.7. Histological Analysis

Testes and epididymides were fixed in Davidson’s fixative (Yuanye Bio-Technology, Shanghai, China, R23045) for 24 h. After 12 h, each testis was cut along its largest transverse plane and returned to the same fixative for an additional 12 h. The tissues were subsequently dehydrated, embedded in paraffin, and cut into 5 μm sections. Testicular and epididymal sections were stained with hematoxylin and eosin (H&E) to evaluate general tissue morphology. Testicular sections were additionally subjected to periodic acid–Schiff (PAS) staining to assess seminiferous epithelial organization and spermatogenic progression. Whole-slide images were acquired using a Pannoramic SCAN II digital slide scanner (3DHISTECH, Budapest, Hungary). Images were examined with Slide Viewer software version 2.6 (3DHISTECH, Budapest, Hungary). For each mouse, at least 20 randomly selected, near-circular seminiferous tubule cross-sections were analyzed. Measurements from these tubules were averaged to obtain one value for each mouse, and the mouse was treated as the biological replicate.

### 2.8. Immunofluorescence Staining

Paraffin sections of the testes were deparaffinized and rehydrated. Antigen retrieval was performed by heating the sections in 1× citrate buffer prepared from a 100× stock solution (Bioss, Beijing, China, C02-02002). The sections were permeabilized with 0.3% Triton X-100 (Sigma-Aldrich, St. Louis, MO, USA, V900502) and subsequently blocked with 10% normal goat serum (Boster, Wuhan, China, AR0009) for 1 h at room temperature. Primary antibodies listed in Table 2 were applied overnight at 4 °C. After three washes with PBS, the sections were incubated for 1 h at room temperature in the dark with Alexa Fluor 488- or 594-conjugated goat anti-mouse IgG (H+L) or goat anti-rabbit IgG (H+L), or Alexa Fluor 647- conjugated donkey anti-goat IgG (H+L) secondary antibodies (Invitrogen, Waltham, MA, USA) at 1:200. For acrosome staining, Alexa Fluor 594-conjugated peanut agglutinin (PNA; Invitrogen, Waltham, MA, USA, L32459) was applied at 1:400 for 1 h at room temperature in the dark [28]. Nuclei were counterstained with DAPI. Images were acquired using an FV3000 confocal laser scanning microscope (Olympus, Tokyo, Japan). Control and CKO sections from the same experiment were imaged using the same acquisition settings.

DDX4^+^ cells were counted manually using ImageJ in 20 randomly selected, near-circular seminiferous tubule cross-sections per mouse. A cell was counted when DDX4^+^ cytoplasm could be assigned to a distinct DAPI-stained nucleus within the seminiferous epithelium. Ambiguous signals that could not be assigned to an individual nucleus were excluded. Counts from the 20 tubules were averaged to obtain one value per mouse.

### 2.9. Computer-Assisted Sperm Analysis (CASA)

Sperm concentration and total motility were measured using computer-assisted sperm analysis (CASA) as previously described [23]. One cauda epididymis from each adult male mouse was collected and cut into small pieces in prewarmed human tubal fluid (HTF) medium (Nanjing Aibei Biotechnology, Nanjing, China, M1135). The final volume of sperm suspension was adjusted to 0.2 mL. Samples were incubated at 37 °C for 8 min to allow sperm to disperse into the medium. The suspension was then transferred to a sperm counting chamber, and sperm concentration and total motility were measured using a WLJY-9000 CASA system (WeiLi Co., Ltd., Beijing, China).

### 2.10. Statistical Analysis

Statistical analyses were conducted using GraphPad Prism 11 (GraphPad Software, Boston, MA, USA). Distribution was assessed with the Shapiro–Wilk test, and equality of variance was evaluated using an F test. When both assumptions were satisfied, differences between two groups were analyzed using a two-tailed unpaired Student’s *t*-test. For animal experiments, each mouse was considered an independent biological replicate. Measurements obtained from multiple seminiferous tubules or microscopic fields were averaged for each mouse before statistical analysis. * *p* < 0.05 was considered statistically significant.

## 3. Results

### 3.1. HSP90α Is Expressed in Mouse Sertoli Cells

**Figure 1:**
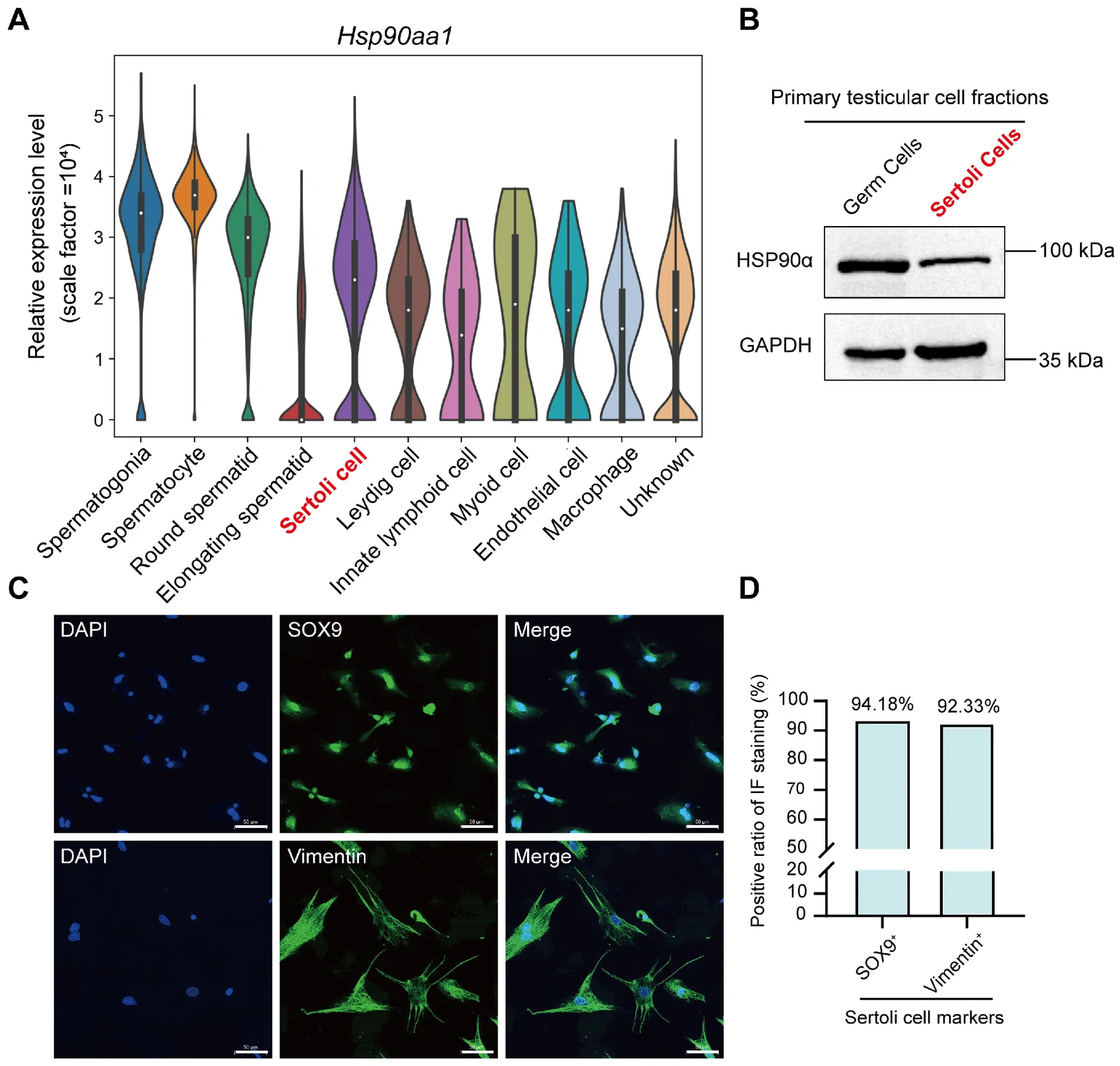
Expression of HSP90α in the mouse testicular cell populations. (**A**) Violin plot of *Hsp90aa1* transcript expression across annotated testicular cell populations, obtained from SMARTdb visualization of dataset GSE112393 [21,22]. The Sertoli-cell label is highlighted in red. (**B**) Representative Western blot showing HSP90α protein in primary testicular cell fractions enriched in germ cells and Sertoli cells. GAPDH served as the loading control. (**C**) Representative immunofluorescence images of SOX9 or vimentin staining in parallel Sertoli cell-enriched cultures. Nuclei were counterstained with DAPI. Scale bars, 50 μm. (**D**) Percentage of SOX9^+^ and vimentin^+^ cells among all DAPI^+^ cells. A total of 1000 cells were counted for each marker.

We examined *Hsp90aa1* transcript expression in testicular cell populations using the SMARTdb visualization of a publicly available single-cell RNA-sequencing dataset [21,22]. A detectable level of *Hsp90aa1* transcripts was present in Sertoli cells (Figure 1A). Among these populations, spermatocytes showed the highest relative transcript level, while spermatogonia and round spermatids also showed relatively high expression. The relative transcript expression in Sertoli cells was lower than that in these germ cell populations. The specificity of HSP90α antibodies was validated in HSP90α-knockout (HSP90α-KO) testes (Figure S1). HSP90α protein was then examined in primary testicular cell fractions. Western blotting detected an HSP90α band in both the germ cell-enriched and Sertoli cell-enriched fractions (Figure 1B). The cellular composition of the Sertoli cell-enriched cultures was evaluated in parallel cultures by separate immunofluorescence staining for SOX9 and vimentin (Figure 1C). SOX9^+^ and vimentin^+^ cells accounted for 94.18% and 92.33% of all DAPI^+^ cells, respectively (Figure 1D). We further examined *Hsp90aa1* mRNA expression in vitro by qPCR using the spermatocyte-derived GC-2 and Sertoli cell-derived TM4 cell lines. Although *Hsp90aa1* mRNA levels were lower in TM4 cells than in GC-2 cells, expression was detectable in TM4 cells (*p* = 0.0355; Figure S2). Together, these findings support the expression of HSP90α in mouse Sertoli cells and provide a rationale for investigating the role of HSP90α in this cell population.

### 3.2. Generation and Validation of Sertoli Cell-Specific Hsp90aa1 Conditional Knockout Mice

**Figure 2:**
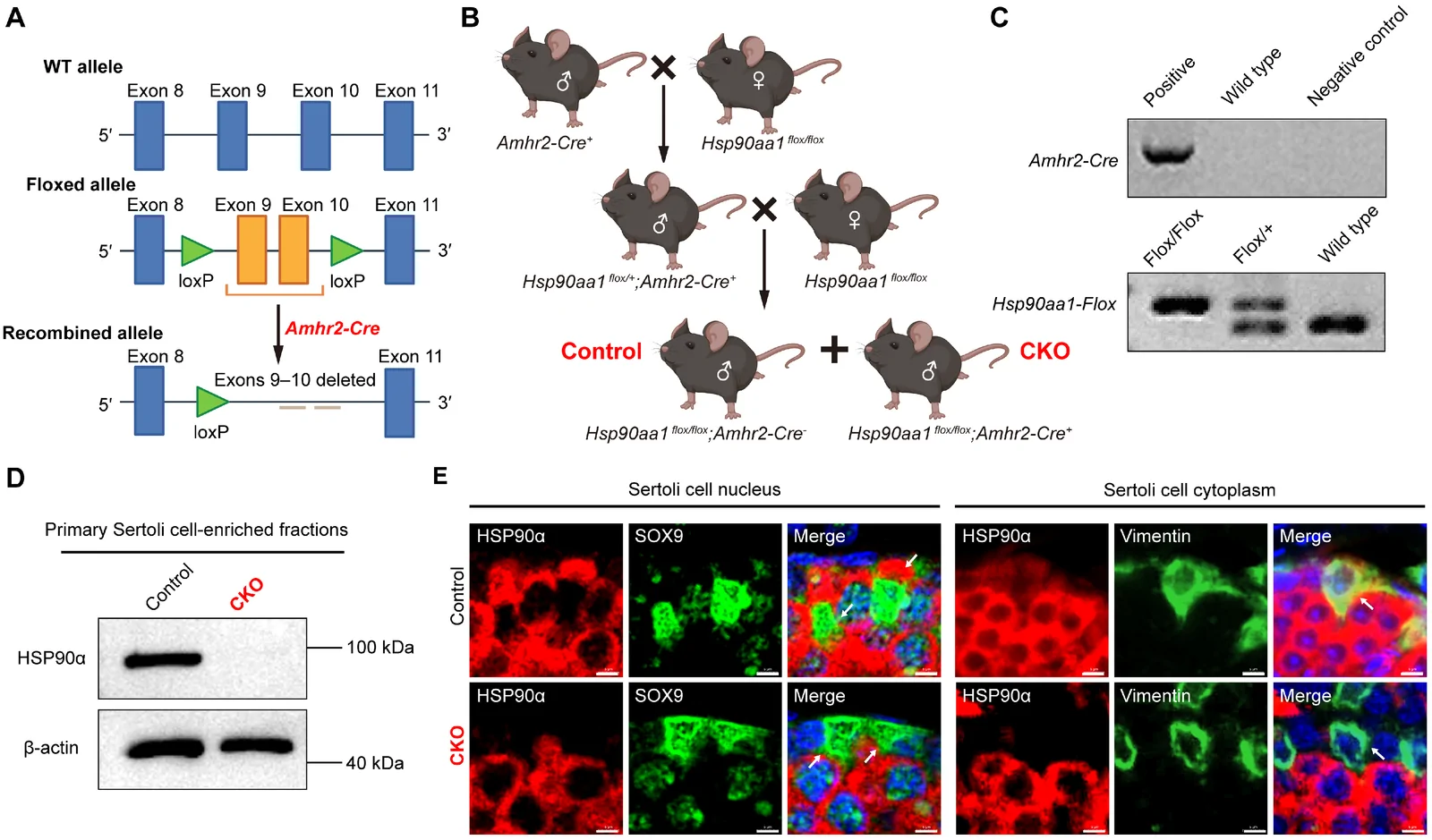
Conditional *Hsp90aa1* allele, breeding strategy, and verification of the CKO model. (**A**) Schematic representation of the wild-type (WT), floxed, and recombined *Hsp90aa1* alleles. Exons 9 and 10 are flanked by loxP sites and are removed after Cre-mediated recombination. (**B**) Breeding strategy used to generate male *Hsp90aa1^flox/flox^*; *Amhr2-Cre*^+^ as CKO mice and *Hsp90aa1^flox/flox^*; *Amhr2-Cre*^−^ littermates as controls. (**C**) PCR genotyping of the *Amhr2-Cre* transgene in the upper panel and the *Hsp90aa1-flox* allele in the lower panel. (**D**) Western blot analysis of HSP90α protein abundance in primary Sertoli cell-enriched fractions obtained from control and CKO testes. β-actin served as the loading control. (**E**) Representative testicular immunofluorescence images of HSP90α with SOX9 or vimentin in 8-week-old control and CKO mice. DAPI marks nuclei, and arrows indicate the examined Sertoli cell regions. Scale bars, 5 μm.

To investigate the physiological role of Sertoli-cell HSP90α in vivo, we generated conditional *Hsp90aa1* knockout mice using *Amhr2*-Cre. The targeted *Hsp90aa1-flox* allele contains loxP sites flanking exons 9 and 10, allowing these exons to be removed through Cre-mediated recombination (Figure 2A). The breeding strategy generated male *Hsp90aa1^flox/flox^*; *Amhr2-Cre*^+^ mice as CKO mice, whereas *Hsp90aa1^flox/flox^*; *Amhr2-Cre*^−^ littermates as controls (Figure 2B). PCR genotyping identified that CKO mice were homozygous for the *Hsp90aa1-flox* allele and positive for the *Amhr2-Cre* transgene (Figure 2C). Western blotting showed a marked reduction in HSP90α protein in primary Sertoli cell-enriched fractions from CKO mice compared with controls (Figure 2D). The enrichment of these fractions was assessed by SOX9 and vimentin staining (Figure S3). We further examined HSP90α in testicular sections using SOX9 and vimentin to identify Sertoli cells (Figure 2E). In control testes, HSP90α was detectable in the cytoplasm surrounding SOX9^+^ Sertoli cell nuclei and overlapped with vimentin^+^ Sertoli cell regions. In CKO testes, the perinuclear HSP90α signal was markedly reduced, and its overlap with vimentin was no longer detectable. Together with the marked protein reduction in Sertoli cell-enriched fractions, these tissue-level findings support successful depletion of HSP90α in Sertoli cells of the CKO mice.

### 3.3. Sertoli Cell-Specific Deletion of Hsp90aa1 Preserves Male Fertility, Relative Testis Weight, or Sperm Parameters

**Figure 3:**
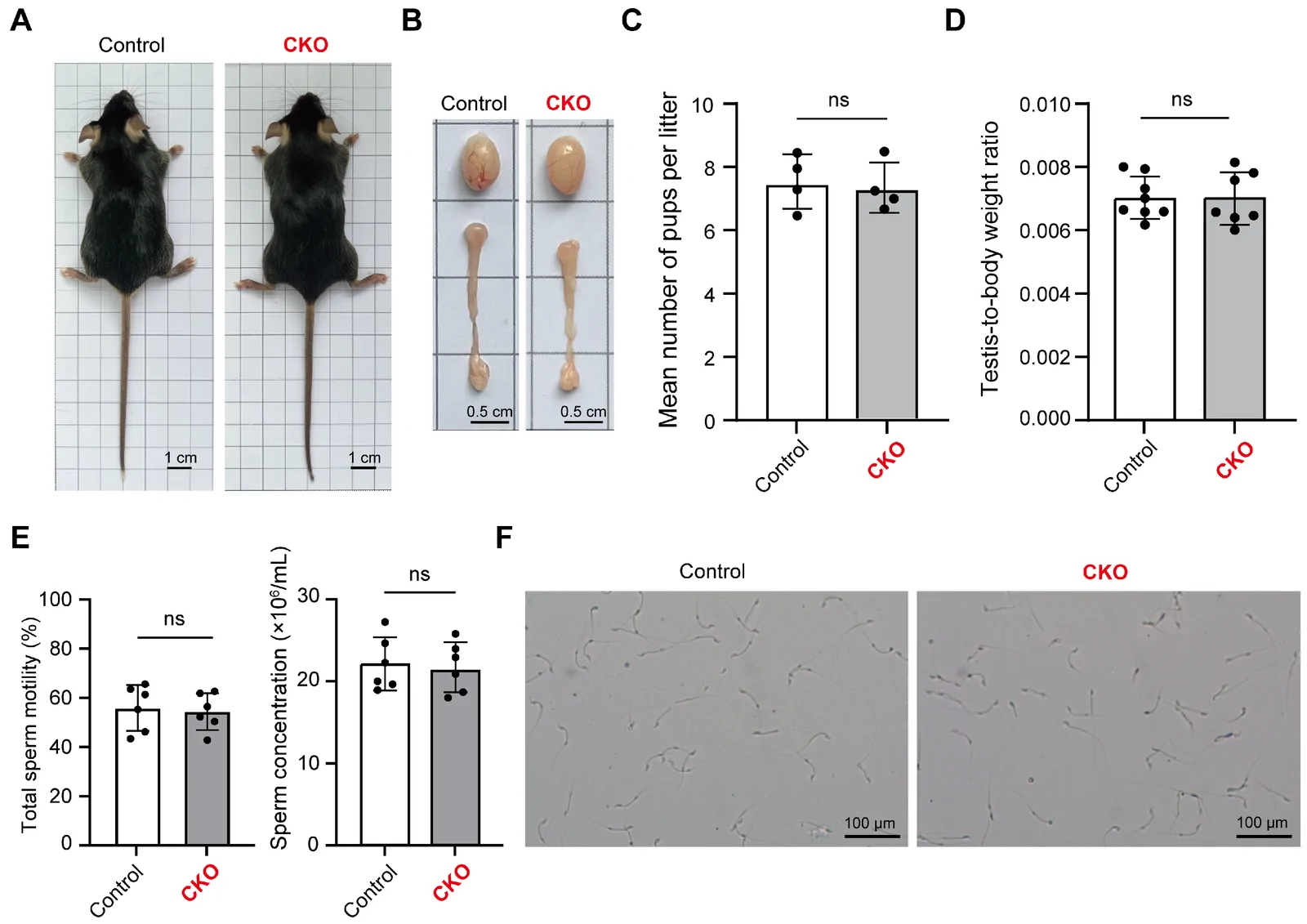
Fertility and sperm parameters of control and CKO males. (**A**) Representative images of adult control and CKO male mice. Scale bars, 1 cm. (**B**) Representative gross images of the testes and epididymides from control and CKO males. Scale bars, 0.5 cm. (**C**) Mean litter size produced by each male during the 2-month fertility assay (n = 4 males per group). (**D**) Testis-to-body weight ratios of adult control and CKO mice (control, n = 8; CKO, n = 7). (**E**) Total sperm motility and concentration of cauda epididymal sperm samples measured by measured by computer-assisted sperm analysis (CASA) (n = 6 males per group). (**F**) Representative bright-field images of cauda epididymal spermatozoa. Scale bars, 100 μm. Each point in (**C**–**E**) represents one biologically independent male. Data are presented as the mean ± SD. Comparisons were performed using an unpaired two-tailed Student’s *t*-test. ns, not significant.

Following validation of the CKO model, we assessed the reproductive phenotype of adult males. Adult CKO males showed general appearance and the gross morphology of their testes and epididymides were similar to those of control littermates (Figure 3A,B). All CKO males included in the 2-month fertility assay sired offspring. The mean number of pups per litter did not differ significantly between control and CKO males, demonstrating that CKO males retained fertility (Figure 3C). Relative testis weight, calculated as the testis-to-body weight ratio, was also comparable between genotypes (Figure 3D). CASA revealed no significant differences in total sperm motility or cauda epididymal sperm concentration (Figure 3E). Representative bright-field images showed a similar overall appearance of cauda epididymal spermatozoa in both groups (Figure 3F). Representative CASA recordings from control and CKO samples are provided in Videos S1 and S2. Thus, CKO males retained fertility without overt changes in the reproductive measures examined under standard laboratory conditions.

### 3.4. Testicular Architecture and Spermatogenic Progression Are Largely Preserved in CKO Mice

**Figure 4:**
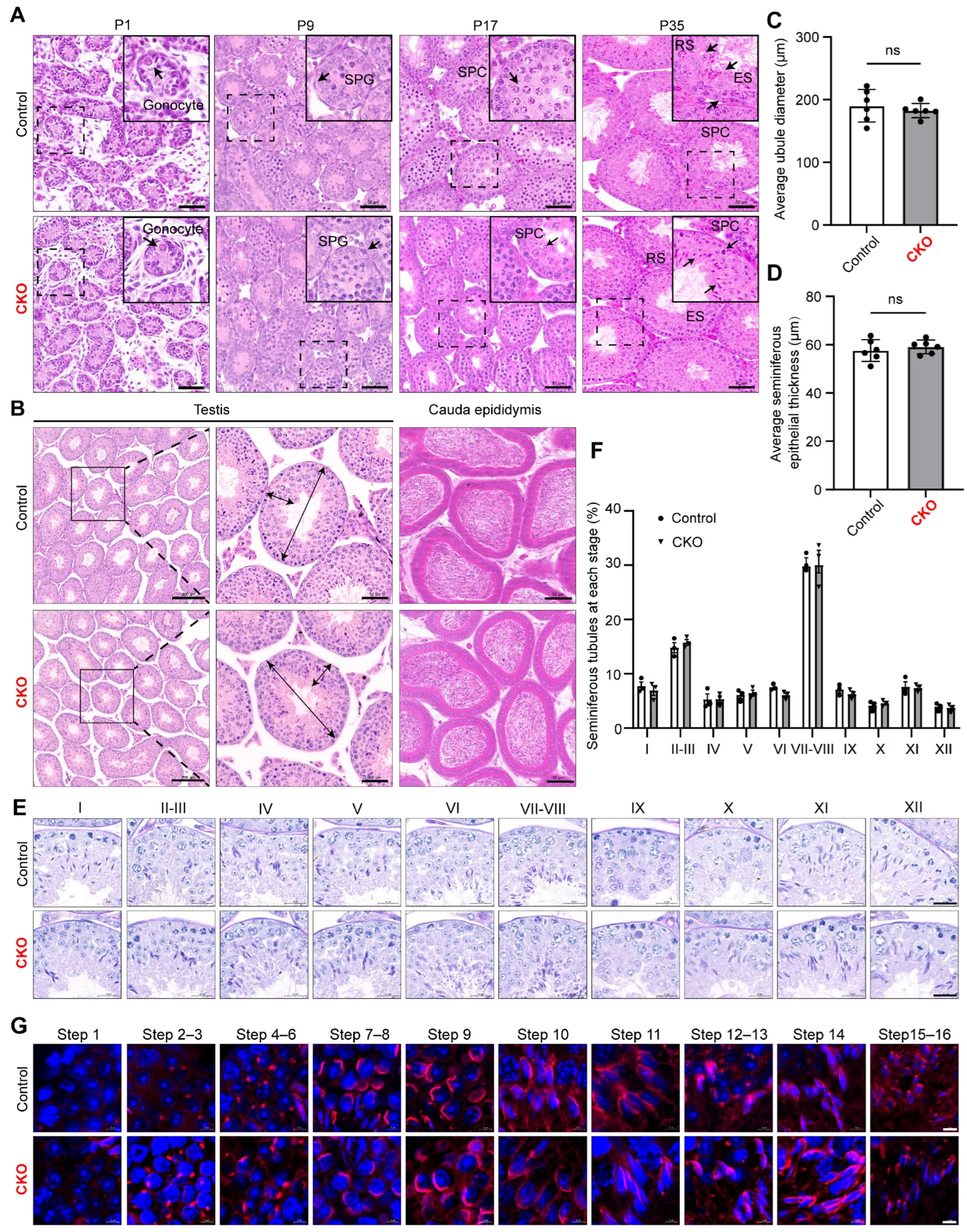
Testicular histology and spermatogenic progression in control and CKO mice. (**A**) Representative hematoxylin and eosin (H&E)-stained testicular sections at P1, P9, P17, and P35. Boxed regions are shown at higher magnification in the insets. Gonocyte, fetal-derived germ cell; SPG, spermatogonium; SPC, spermatocyte; RS, round spermatid; ES, elongated spermatid. Scale bars, 50 μm. (**B**) Representative H&E-stained sections of adult testes and cauda epididymides. Boxed testicular regions are enlarged in the adjacent panels. Arrows indicate the measurements of seminiferous tubule diameter and epithelial thickness. Scale bars, 200 μm in the low-magnification images and 50 μm in the enlarged images. (**C**,**D**) Mean seminiferous tubule diameter (**C**) and seminiferous epithelial thickness (**D**). Twenty near-circular tubule cross-sections were measured per mouse, and the measurements were averaged to obtain one value per mouse (*n* = 6 mice per group). (**E**) Representative periodic acid–Schiff (PAS)-stained seminiferous tubules assigned to the indicated stage groups. Scale bars, 20 μm. (**F**) Proportions of seminiferous tubules in each stage group. Approximately 200 tubule cross-sections were staged per ouse (*n* = 3 mice per group), and one proportion for each stage group was calculated per mouse. (**G**) Representative peanut agglutinin (PNA) staining at the indicated steps of spermiogenesis. PNA-labeled acrosomes are shown in red and nuclei in blue. Scale bars, 5 μm. Quantitative data are presented as mean ± SD; each point represents one mouse. Unpaired two-tailed *t*-tests were used for (**C**,**D**), and multiple unpaired two-tailed *t*-tests for (**F**). ns, not significant.

To examine testicular development after Sertoli cell-specific *Hsp90aa1* deletion, we compared H&E-stained sections from control and CKO mice at P1, P9, P17, and P35. At each age, the two groups showed broadly similar seminiferous cord or tubule organization and the expected age-associated appearance of developing germ cells (Figure 4A). In adult testes, both groups displayed organized seminiferous tubules containing germ cells at successive stages of development. Spermatozoa were also present in the cauda epididymal lumina (Figure 4B; Figure S4 shows H&E sections from three individual mice per group). Mean seminiferous tubule diameter and epithelial thickness did not differ significantly between groups (Figure 4C,D). We next assessed the seminiferous epithelial cycle by PAS staining. Tubules representing the examined stages were identified in both groups (Figure 4E), and the proportions assigned to each stage group did not differ significantly between control and CKO testes (Figure 4F). PNA staining showed the expected changes in acrosomal morphology across the illustrated steps of spermiogenesis in both groups (Figure 4G). Together, these observations show no major disruption of testicular architecture or the assessed progression of spermatogenesis in CKO mice.

### 3.5. Sertoli Cell-Specific Deletion of Hsp90aa1 Modestly Reduces Germ Cell Counts Without Affecting Sertoli Cell Counts

**Figure 5:**
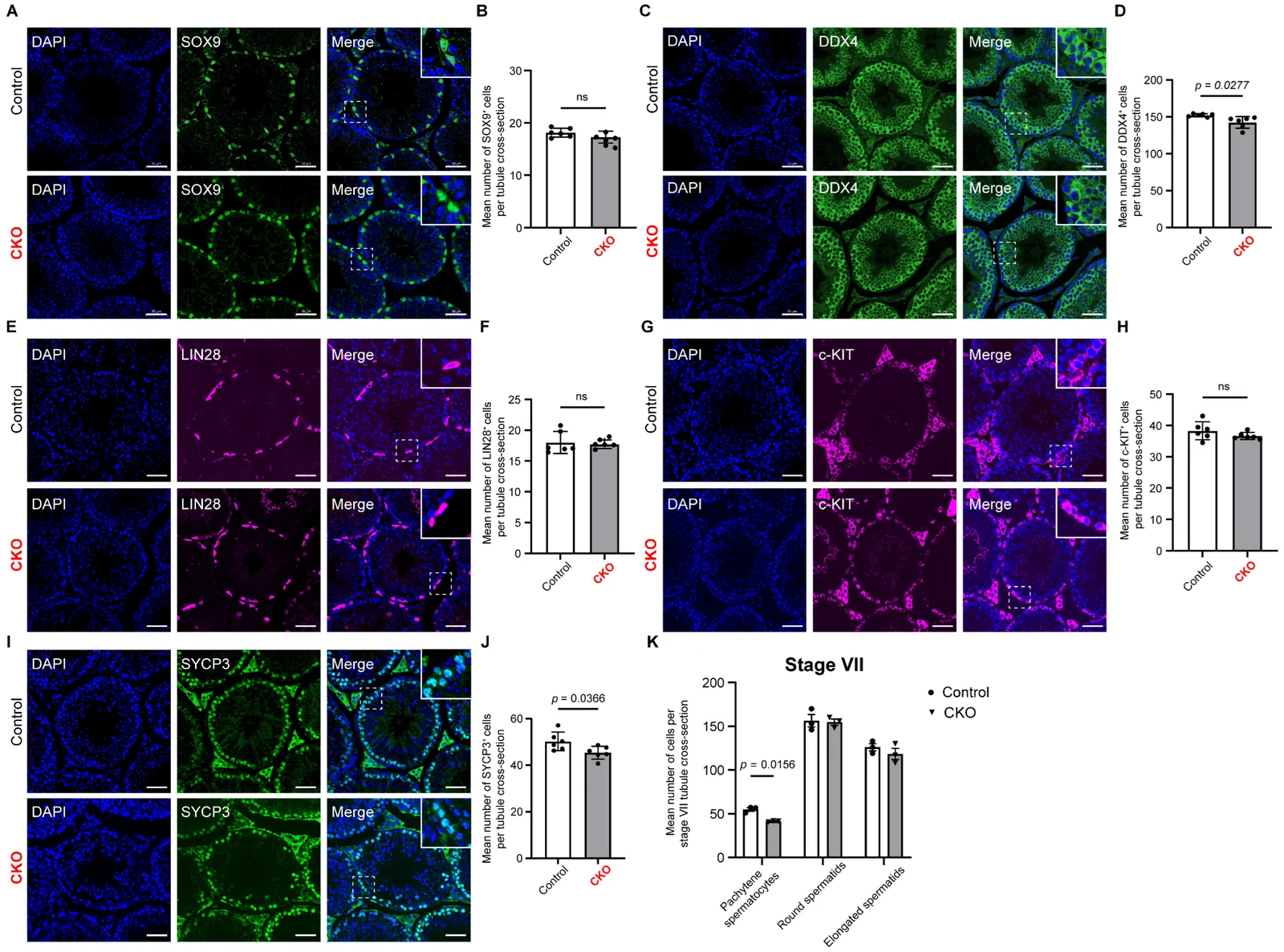
Sertoli cell and germ cell populations in adult control and CKO testes. (**A**,**C**,**E**,**G**,**I**) Representative immunofluorescence images of testicular sections stained for SOX9 (**A**), DDX4 (**C**), LIN28 (**E**), c-KIT (**G**), or SYCP3 (**I**), with nuclei counterstained using DAPI. Boxed regions are shown at higher magnification in the corresponding insets to show the cellular localization of each marker more clearly. (**B**,**D**,**F**,**H**,**J**) Mean numbers of SOX9^+^ Sertoli cells (**B**), DDX4^+^ germ cells (**D**), LIN28^+^ cells (**F**), c-KIT^+^ cells (**H**), and SYCP3^+^ cells (**J**) per seminiferous tubule cross-section (*n* = 6 mice per group). (**K**) Mean counts of pachytene spermatocytes, round spermatids, and elongated spermatids per stage VII tubule cross-section (*n* = 3 mice per group). Each point represents one mouse. Data are presented as mean ± SD. Unpaired two-tailed Student’s *t*-tests were used for (**B**,**D**,**F**,**H**,**J**), and multiple unpaired two-tailed *t*-tests were used for (**K**). The indicated comparisons yielded *p* = 0.0277 (**D**), *p* = 0.0366 (**J**), and *p* = 0.0156 for pachytene spermatocytes (**K**); ns, not significant. Scale bars, 50 μm.

We examined Sertoli cells and germ cell populations in adult testicular sections. SOX9^+^ Sertoli cell nuclei retained their expected basal position, and their mean count per seminiferous tubule cross-section did not differ significantly between control and CKO mice (Figure 5A,B). In contrast, the mean number of DDX4^+^ germ cells per tubule cross-section was modestly lower in CKO testes (*p* = 0.0277; Figure 5C,D).

To identify the germ cell population associated with this difference, we examined markers of spermatogonia and spermatocytes. Counts of LIN28^+^ and c-KIT^+^ spermatogonia did not differ significantly between groups (Figure 5E–H). The mean SYCP3^+^ cell count was modestly reduced in CKO testes (*p* = 0.0366; Figure 5I,J). Because cell composition varies with seminiferous epithelial stage, we also counted morphologically identified cells in stage VII tubules. Pachytene spermatocyte counts were lower in CKO mice (*p* = 0.0156), whereas round and elongated spermatid counts did not differ significantly between groups (Figure 5K). These findings associate the modest reduction in total germ cell counts primarily with the spermatocyte population, without a detectable reduction in the examined spermatogonial or spermatid populations.

### 3.6. Basal Localization of ZO-1 and Occludin Is Retained in CKO Testes

**Figure 6:**
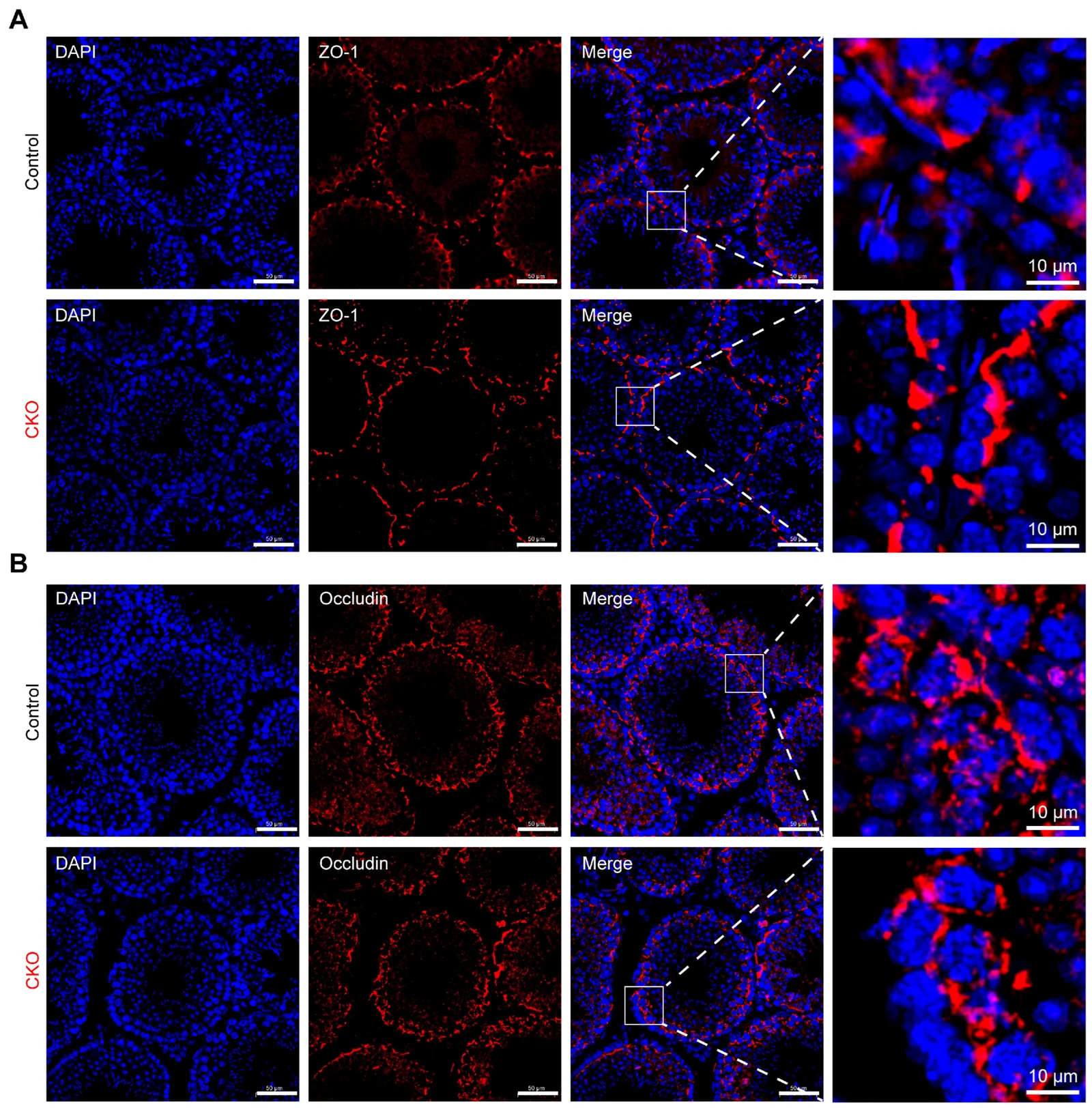
Localization and distribution of junction-associated proteins in control and CKO testes. (**A**,**B**) Representative immunofluorescence images of adult control and CKO testicular sections stained for ZO-1 (**A**, red) or occludin (**B**, red), with nuclei counterstained using DAPI in blue. Boxed regions in the merged images are presented at higher magnification in the rightmost panels. Control and CKO images were acquired and processed using identical settings. Scale bars, 50 μm in the overview images and 10 μm in the enlarged images.

We next examined whether Sertoli cell-specific *Hsp90aa1* deletion altered the localization of junction-associated proteins. ZO-1 is a cytoplasmic scaffolding protein located at the intracellular face of tight junctions, where it links transmembrane junctional proteins, including occludin, to the actin cytoskeleton. Occludin is an integral transmembrane protein that contributes to Sertoli cell tight junction barrier function [29,30]. Immunofluorescence staining showed that ZO-1 was concentrated near the basal region of the seminiferous epithelium in both control and CKO testes. The signal formed comparable continuous patterns along the basal compartment in the two groups (Figure 6A). Enlarged images confirmed that the overall localization and distribution of ZO-1 were maintained in CKO testes. Occludin displayed a similar basal distribution in control and CKO seminiferous tubules (Figure 6B). Thus, the observed localization patterns of ZO-1 and occludin were retained following Sertoli cell-specific *Hsp90aa1* deletion.

## 4. Discussion

The present study examined the contribution of Sertoli cell HSP90α to male reproduction using an *Amhr2*-Cre-mediated conditional *Hsp90aa1* knockout model. HSP90α was markedly reduced in Sertoli cell-enriched fractions, and testicular staining showed reduced signal in marker-identified Sertoli cell regions. Under the conditions examined, CKO males retained fertility, and no major changes were detected in the measured sperm parameters, postnatal testicular architecture, or distribution of seminiferous epithelial stages. Our cell-specific analysis *in vivo* helps distinguish the contribution of Sertoli cell HSP90α from the severe reproductive phenotype previously reported after systemic *Hsp90aa1* loss [3,4].

The milder phenotype in our Sertoli cell CKO model suggests that loss of HSP90α from this compartment alone does not reproduce those severe defects. At the same time, the reduction in spermatocyte counts indicates that Sertoli cell HSP90α may contribute to the environment supporting this germ cell population. The current study does not identify the direct cause of the reduction. The agreement between the overall SYCP3^+^ count and the stage VII pachytene spermatocyte count strengthens the interpretation that spermatocytes contribute to the modest DDX4^+^ cell reduction. Stage-matched counting also reduces the influence of differences in epithelial stage distribution on this comparison. Neither round nor elongated spermatid counts differed significantly in the stage VII analysis, and the measured sperm parameters and fertility were retained. Testicular spermatocyte counts reflect the cellular composition at the time of sampling, whereas the measured sperm parameters and fertility reflect reproductive outcomes over time. These results indicate that the cellular change observed at eight weeks was not accompanied by a detectable decline in the downstream reproductive outcomes measured here.

One possible explanation for the modest reduction in spermatocytes is altered metabolic support from Sertoli cells. A study of cultured immature porcine Sertoli cells linked *HSP90AA1* to lactate production [20], while Sertoli-cell-derived lactate serves as an energy substrate for developing germ cells, including spermatocytes and spermatids [31]. Reduced lactate support may therefore contribute to the lower spermatocyte counts observed in our CKO mice. Because lactate production was not measured in this study, this possibility remains to be tested. Whether altered metabolic support contributes to the reduction in spermatocytes therefore remains to be tested. Differences in species, developmental stage, and experimental setting may also help explain why the marked effects reported in cultured cells were not reflected in a major fertility phenotype in our model.

ZO-1 and occludin retained their observed basal localization patterns in CKO testes. This finding supports preservation of the localization of the two proteins examined, but it does not establish normal BTB permeability. A functional tracer assay would be needed to test barrier permeability directly. Although tissue staining and Western blotting of Sertoli cell-enriched fractions support marked HSP90α reduction in Sertoli cells, we did not directly determine the extent of *Amhr2*-Cre-mediated recombination among Sertoli cells. Therefore, we cannot establish that every Sertoli cell was targeted. The postnatal histology and adult Sertoli cell counts did not reveal an overt developmental defect, although embryonic Sertoli cell proliferation and survival were not directly examined and an early or transient effect cannot be excluded. The absence of major abnormalities under standard housing conditions also does not exclude a greater requirement for HSP90α during stress. Since HSP90α is stress-responsive, future work could assess whether Sertoli cell *Hsp90aa1* deletion alters germ cell support or junctional organization under defined challenges.

In summary, Sertoli cell-specific *Hsp90aa1* deletion was associated with a modest reduction in spermatocyte counts but did not overtly impair male fertility under the conditions examined. These in vivo findings distinguish the reproductive outcome of Sertoli cell deletion from that reported after systemic *Hsp90aa1* loss and support a cell type-dependent contribution of HSP90α to male reproduction.

## 5. Conclusions

Using an *Amhr2*-Cre-mediated conditional knockout model, we found that Sertoli cell-specific deletion of *Hsp90aa1* was associated with a modest reduction in spermatocyte counts. Nevertheless, CKO males retained fertility under the conditions examined, with no major abnormalities in the measured sperm parameters, testicular histology, or distribution of seminiferous epithelial stages. The basal localization of ZO-1 and occludin was also retained. These findings indicate that Sertoli cell *Hsp90aa1* deletion does not overtly impair male fertility under standard laboratory conditions, although it is associated with a subtle change in the spermatocyte population.

## Figures and Tables

**Table 1:** Primer sequences used for genotyping and RT-qPCR.

Application	Gene	Primer Sequence (5′-3′)
Genotyping	*Hsp90aa1-Flox*	F: GCTTTCAGAGCTGTTGCGG
		R: CAACCTGGTCCTTGGTCTCAC
	*Amhr2-Cre*	F: TTGCCTGCATTACCGGTCGATGC
		R: TTGCACGTTCACCGGCATCAACG
RT-qPCR	*Hsp90aa1*	F: AATTGCCCAGTTAATGTCCTTGA
		R: CGTCCGATGAATTGGAGATGAG
	*Gapdh*	F: AGGTCGGTGTGAACGGATTTG
		R: GGGGTCGTTGATGGCAACA

**Table 2:** List of primary antibodies used in this study.

Antibody	Company, Cat. No	Host Species	Application (Dilution)
SOX9	Millipore (Burlington, MA, USA), AB5535	Rabbit	IF (1:200)
Vimentin	Servicebio (Wuhan, China), GB11192-50	Rabbit	IF (1:200)
HSP90α	GL Biochem (Shanghai, China), custom-generated	Rabbit	WB (1:1000); IF (1:200)
HSP90α	Abcam (Cambridge, UK), ab79849	Mouse	WB (1:1000)
GAPDH	ABclonal (Wuhan, China), AC033	Mouse	WB (1:10,000)
β-actin	ABclonal (Wuhan, China), AC050	Rabbit	WB (1:10,000)
DDX4	Abcam (Cambridge, UK), ab27591	Mouse	IF (1:200)
LIN28	R&D Systems (Minneapolis, MN, USA), AF3757	Goat	IF (1:200)
c-KIT	R&D Systems (Minneapolis, MN, USA), AF1356	Goat	IF (1:200)
SYCP3	Santa Cruz (Dallas, TX, USA), sc-74569	Mouse	IF (1:200)
ZO-1	ABclonal (Wuhan, China), A11417	Rabbit	IF (1:200)
Occludin	Abcam (Cambridge, UK), ab242202	Mouse	IF (1:200)

## Data Availability

All relevant data are included in the article and its Supplementary Materials.

## Supplementary Materials

The following supporting information can be downloaded at https://www.mdpi.com/article/10.3390/cells15211962/s1. Figure S1: Validation of HSP90α antibodies using wild-type (WT) and HSP90α-knockout (HSP90α-KO) testes; Figure S2: *Hsp90aa1* mRNA expression in GC-2 and TM4 cells; Figure S3: Purity assessment of the Sertoli cell-enriched fractions analyzed in Figure 2D; Figure S4: Testicular histology of individual control and CKO mice; Video S1: Representative CASA recording of cauda epididymal sperm from a control male; Video S2: Representative CASA recording of cauda epididymal sperm from a CKO male.
