# Supplementary material for: Sertoli Cell-Specific Deletion of Hsp90aa1 Does Not Overtly Impair Male Fertility in Mice
Source: Cells. 2026 Oct 28;15(21):1962. doi: 10.3390/cells15211962 (PMC13762123; doi:10.3390/cells15211962)
Supplement: Supplementary file 1 [file cells-15-01962-s001.zip › cells-4596852-Uncropped Western blot images.pdf]

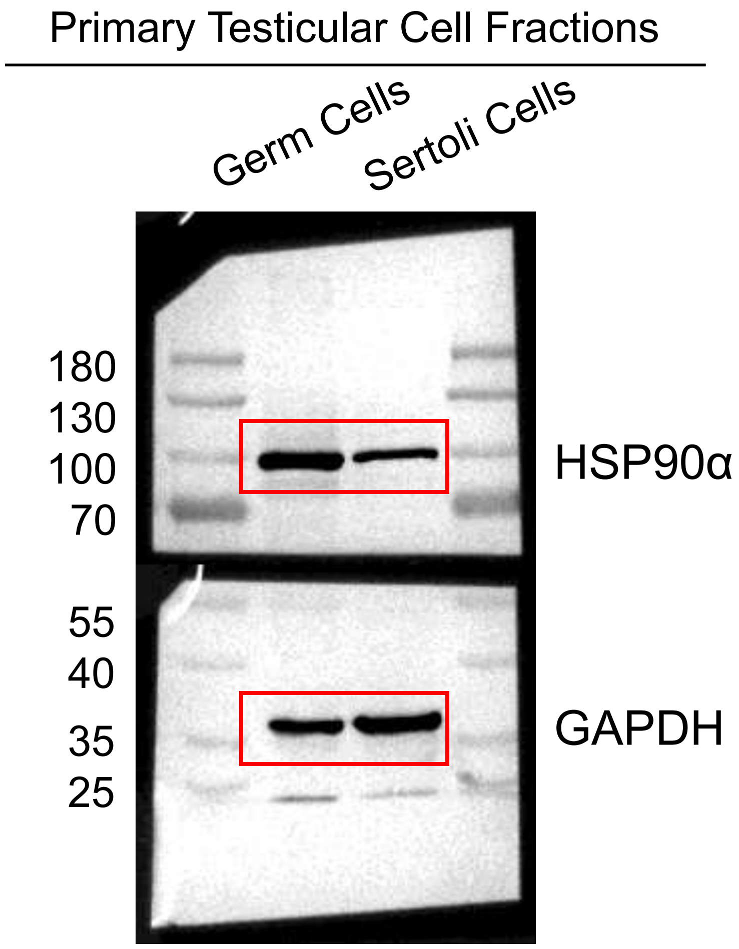

Figure 1B

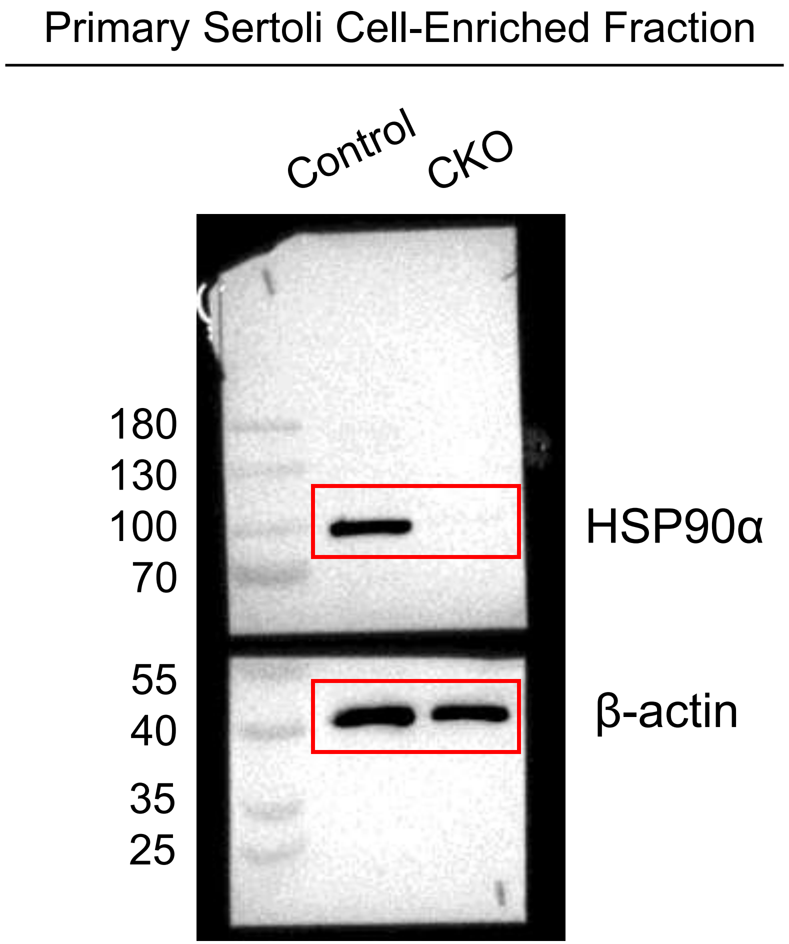

Figure 2D

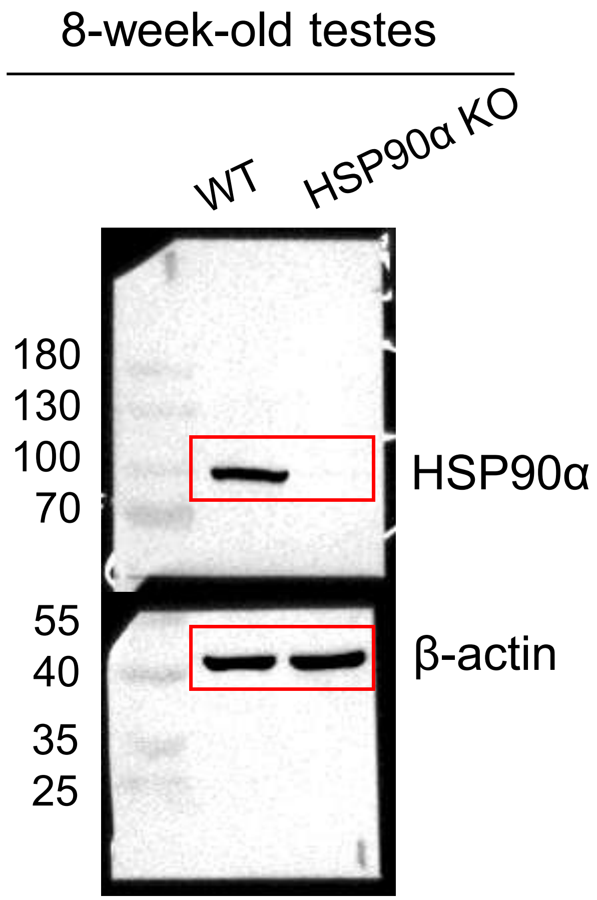

Custom-generated rabbit antibody
(GL Biochem)

Figure S1A

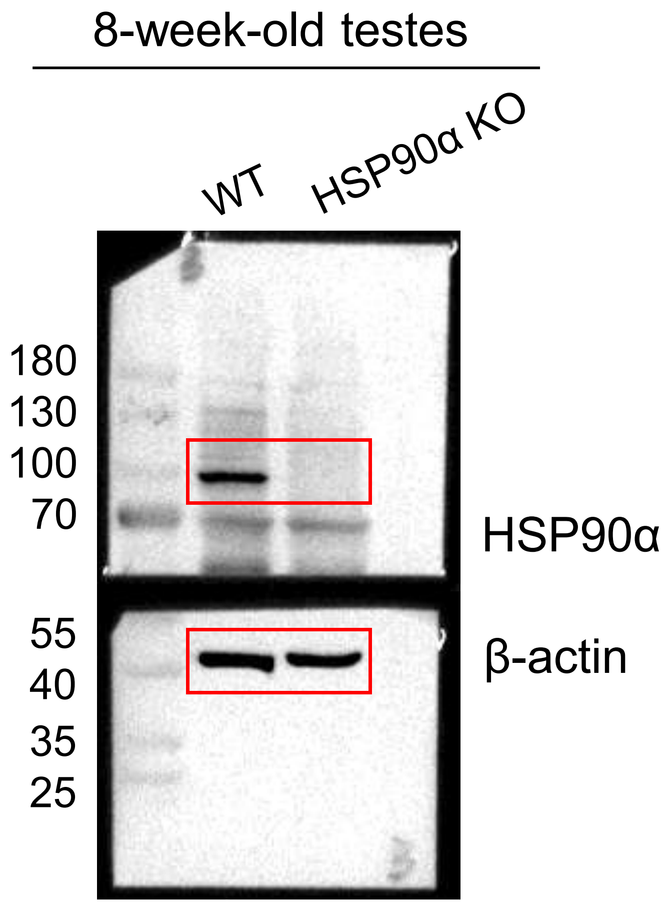

Commercial mouse antibody
(Abcam, ab79849)

Figure S1B
