# Supplementary material for: Sertoli Cell-Specific Deletion of Hsp90aa1 Does Not Overtly Impair Male Fertility in Mice
Source: Cells. 2026 Oct 28;15(21):1962. doi: 10.3390/cells15211962 (PMC13762123; doi:10.3390/cells15211962)
Supplement: Supplementary file 1 [file cells-15-01962-s001.zip › Supplementary Figures S1-S4.pdf]

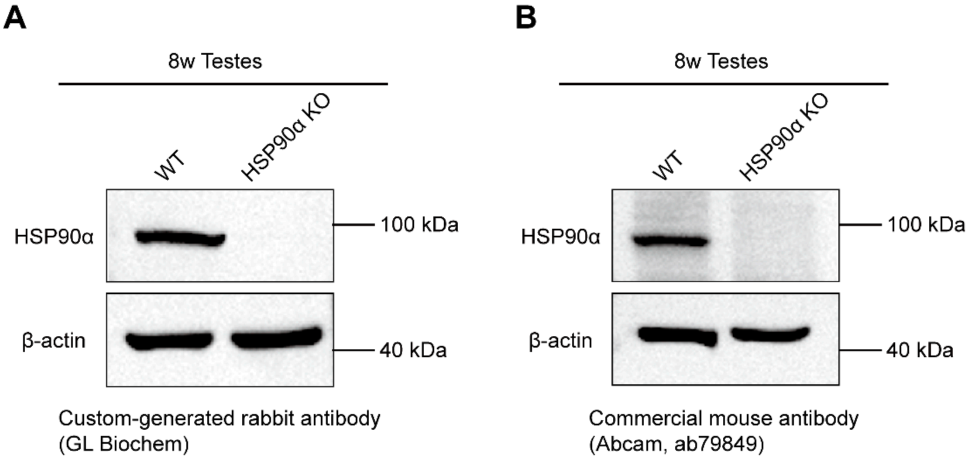

Figure S1. Validation of HSP90α antibodies using wild type (WT) and HSP90α-knockout (HSP90α-KO) testes. Western blot analysis of testicular lysates from 8-week-old WT and HSP90α-KO mice (Shanghai Model Organisms Center, Shanghai, China, NM-KO-190731) using **(A)** a custom-generated rabbit anti-HSP90α antibody from GL Biochem and **(B)** a commercial mouse anti-HSP90α antibody from Abcam (ab79849). β-Actin was used as a loading control.

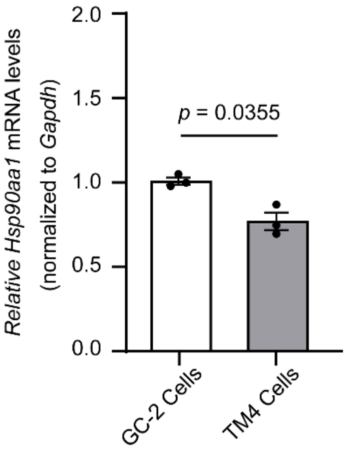

Figure S2. *Hsp90aa1* mRNA expression in GC-2 and TM4 cells. *Hsp90aa1* mRNA levels were measured by quantitative real-time PCR, normalized to *Gapdh*, and expressed relative to GC-2 cells. Data are presented as mean ± SEM. Statistical significance was assessed using an unpaired two-tailed Student's *t*-test; p = 0.0355.

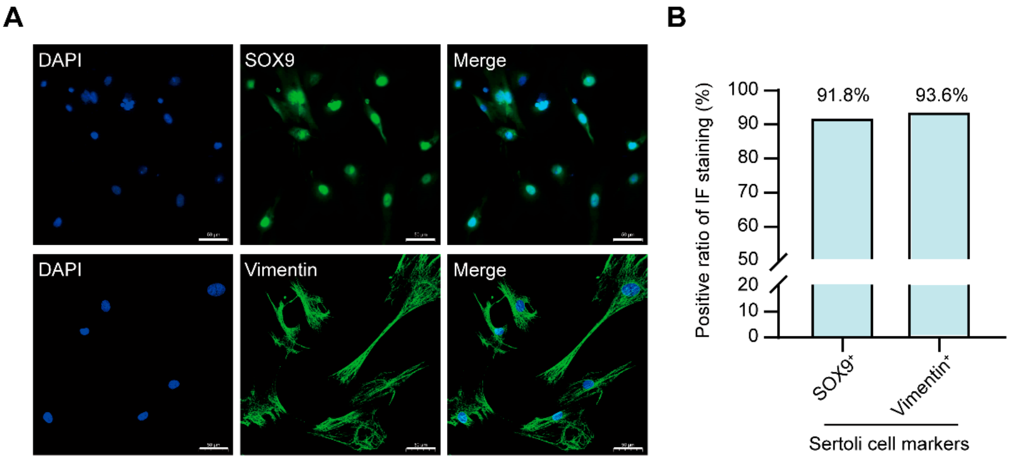

Figure S3. Purity assessment of the Sertoli cell-enriched fractions analyzed in Figure 2D. **(A)** Representative immunofluorescence images of the cultured primary cell fractions used for the Western blot analysis in Figure 2D, stained for SOX9 or vimentin, with nuclei counterstained using DAPI. Scale bars, 50 μm. **(B)** Percentages of $SOX9^+$ and $vimentin^+$ cells among DAPI-stained cells, calculated separately for each marker. A total of 1,000 cells were evaluated for each marker.

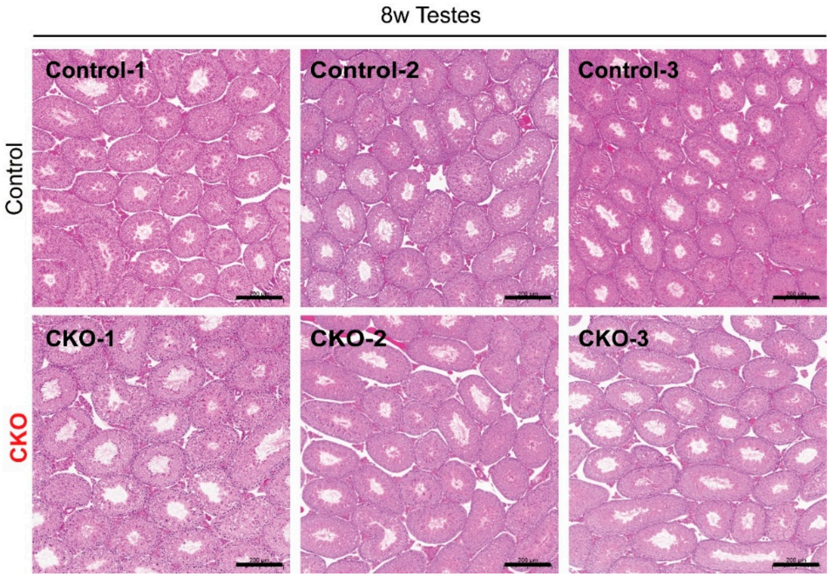

Figure S4. Testicular histology of individual control and CKO mice. H&E-stained testicular sections from three individual control mice and three individual CKO mice at 8 weeks of age. Each panel represents a different mouse. Scale bars, 200 μm.
